# Low-Grade Appendiceal Mucinous Neoplasm With Discontinuous Mucin Infiltration Into the Cecal Wall: A Case Report

**DOI:** 10.7759/cureus.106609

**Published:** 2026-04-07

**Authors:** Nariyasu Tabara, Yuu Otani, Yoshida Seiya, Tetsu Nakamura

**Affiliations:** 1 Division of Gastroenterological Surgery, Hyogo Cancer Center, Kobe, JPN; 2 Division of General Surgery, Izumo Tokushukai Hospital, Izumo, JPN

**Keywords:** appendiceal cancer, appendiceal tumor, cecal wall infiltration, low-grade appendiceal mucinous neoplasm, mucinous neoplasm

## Abstract

Low-grade appendiceal mucinous neoplasm (LAMN) can lead to intraperitoneal dissemination and subsequent pseudomyxoma peritonei (PMP). Therefore, achieving complete resection, typically by appendectomy, is considered important for curative treatment. We report a case in which discontinuous mucin infiltration into the cecal wall was identified only after secondary resection, providing insight into the extent of surgical resection. To the best of our knowledge, such cases appear to be rare, with limited reports in the literature.

A 60-year-old female was incidentally found to have an enlarged appendix during evaluation for pneumonia. She underwent a laparoscopic appendectomy, and histopathological examination led to a diagnosis of LAMN. Based on the pathological findings, additional laparoscopic ileocecal resection was recommended, and the patient provided informed consent. Histological examination of the resected specimen revealed discontinuous acellular mucin infiltration limited to the muscularis propria of the cecal wall, which had not been detected preoperatively or intraoperatively. Surgical margins were negative, and no epithelial components were identified. No recurrence was observed during a two-year follow-up period.

LAMN requires complete resection to reduce the risk of progression to PMP, which is associated with a poorer prognosis. Lymph node metastasis is rare, and limited resection is generally considered sufficient. However, in the present case, discontinuous mucin infiltration was identified within the cecal wall despite negative surgical margins. This finding highlights the potential limitation of preoperative imaging and intraoperative assessment in detecting such lesions. One possible speculative mechanism is increased intraluminal pressure due to mucin retention, which may lead to mucosal disruption or vascular permeation, allowing mucin to infiltrate the wall. However, this hypothesis remains speculative and requires further investigation.

In LAMN cases with submucosal or deeper involvement, the extent of resection may warrant careful consideration, as discontinuous mucin infiltration may occur. These findings are hypothesis-generating, and further studies are needed to clarify the optimal surgical strategy.

## Introduction

Low-grade appendiceal mucinous neoplasm (LAMN) has been newly classified in the Japanese Classification of Colorectal, Appendiceal, and Anal Carcinoma, 8th Edition [1]. In the WHO classification, LAMN is defined as a low-grade appendiceal mucinous neoplasm characterized by low-grade cytologic atypia and pushing growth, corresponding to a carcinoma in situ rather than an invasive carcinoma [2]. Complete resection is essential, as mucin-producing epithelial components may disseminate into the peritoneal cavity, potentially leading to pseudomyxoma peritonei (PMP). In general, appendectomy is considered sufficient for curative treatment in cases confined to the appendix.

We recently encountered a case of LAMN with discontinuous mucin infiltration within the cecal wall extending beyond the submucosal layer. Such discontinuous infiltration may have implications for determining the appropriate extent of resection. To the best of our knowledge, similar cases appear to be rare, with limited reports describing discontinuous mucin infiltration identified after resection. This case is reported in accordance with the SCARE (Surgical CAse REport) checklist [3].

## Case presentation

In August 2022, a 60-year-old female with cough and fever presented to our department and was diagnosed with pneumonia based on computed tomography (CT) findings. At that time, her appendix was found to be enlarged, and she was referred to our department after treatment for pneumonia. Her medical history was unremarkable, with no known family history of colon cancer. Laboratory blood tests revealed no abnormalities such as anemia or renal dysfunction.

**Figure 1:**
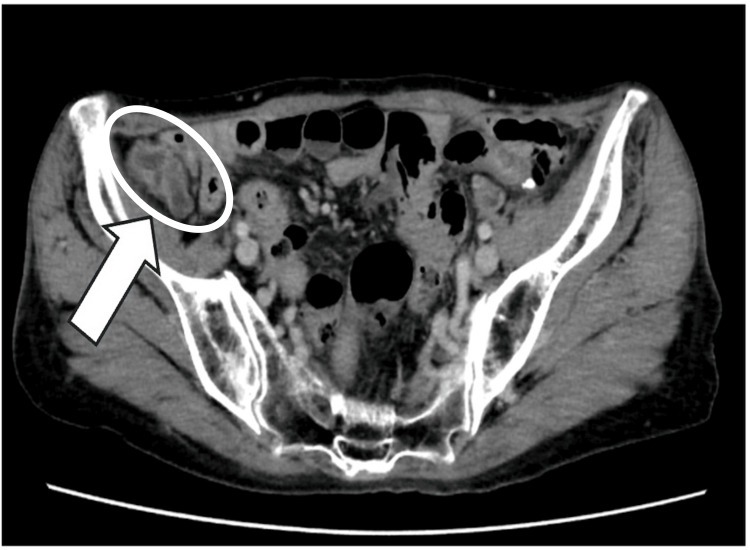
Abdominal computed tomography findings. Abdominal computed tomography revealed an enlarged appendix measuring approximately 13 mm in diameter with a thickened appendiceal wall. The area of interest is indicated by an arrow.

Abdominal CT revealed an enlarged appendix, measuring 13 mm, with evidence of a thickened appendix wall. The appendiceal lumen demonstrated low attenuation on CT, consistent with mucin accumulation. No calcification, wall thickening, or mural irregularity was observed in the appendix (Figure 1).

Preoperative colonoscopy revealed a normal-appearing appendiceal orifice, with no evidence of bulging or mucus discharge, and no other noteworthy findings in the colon. Based on these findings, an elective laparoscopic appendectomy was performed after treatment for pneumonia due to the diagnosis of an appendiceal tumor, particularly LAMN.

Surgical findings were as follows: the appendix was enlarged, and cystic lesions were observed at the base, congregating toward the peripheral side. There were no clear ascites, intraperitoneal dissemination, or liver metastasis. Although the resection range was close to the base of the appendix, en bloc resection was performed using a linear stapler 10 mm toward the cecal side from the base to sufficiently secure the surgical margin distance from the cystic lesion.

**Figure 2:**
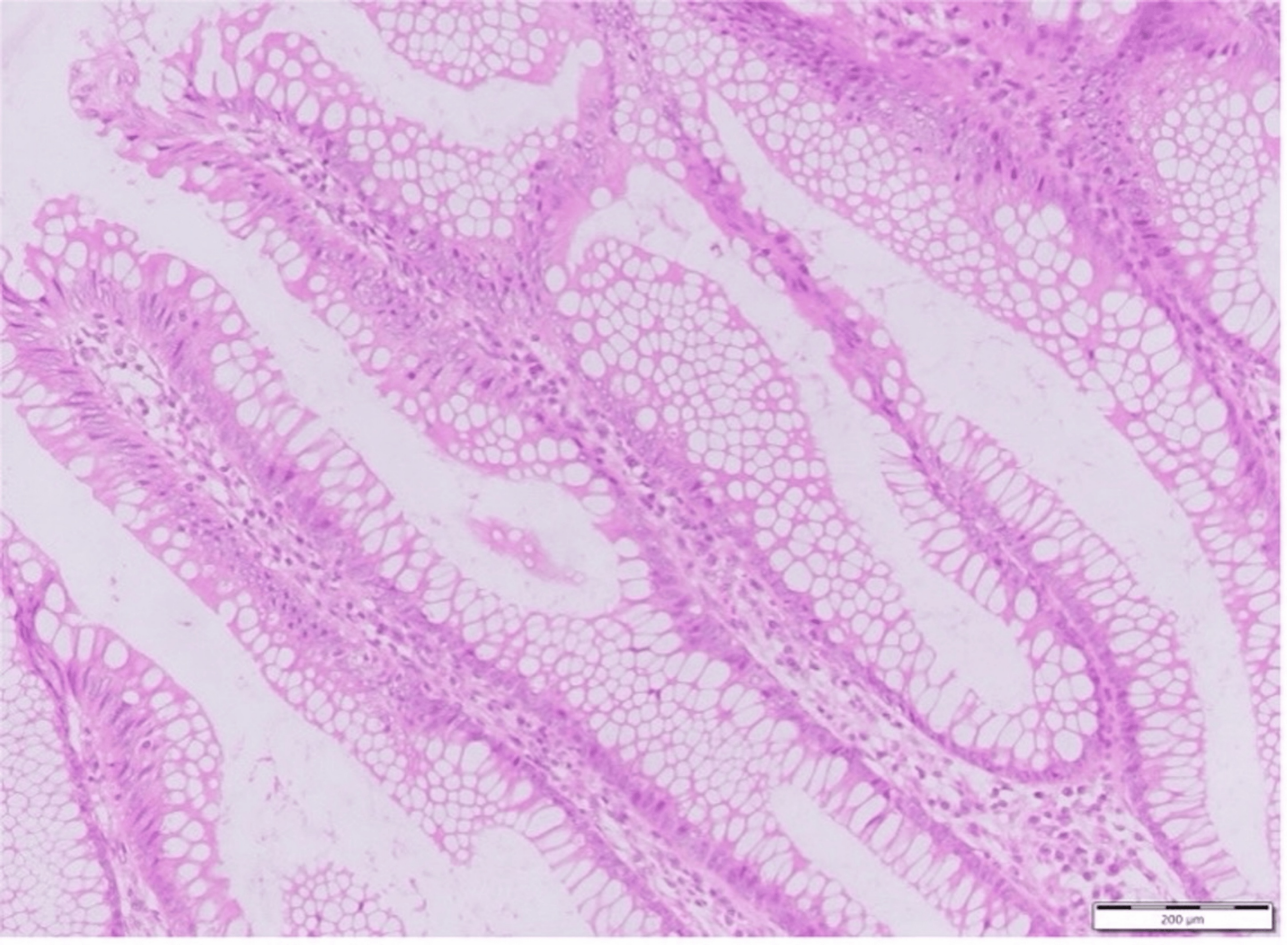
Hematoxylin and eosin (H&E) staining (400x) of the sample collected during the first surgery. Mucus-producing mildly atypical epithelium exhibited pathological findings consistent with low-grade appendiceal mucinous neoplasm (LAMN), with papillary proliferation.

Histopathological examination revealed papillary proliferation of the epithelium showing low-grade atypia, accompanied by mucus production. Mucus masses were observed partially in the subserosal layer. No destructive invasion was observed (Figure 2). Pathological diagnosis was LAMN pT3N0M0.

The patient recovered well and was discharged on postoperative day six. The pathological results were explained to the patient during an outpatient visit. She was provided with an explanation of her diagnosis with LAMN according to the WHO classification. Given the pathological findings (pT3) and the potential risk of residual disease or further extension, additional ileocecal resection was recommended, and the patient subsequently provided informed consent.

Laparoscopic ileocecal resection was subsequently conducted. Intraoperative findings revealed that the previous surgery had caused adhesion between the ileocecum and the abdominal wall, although with no disseminated lesions or ascites.

**Figure 3:**
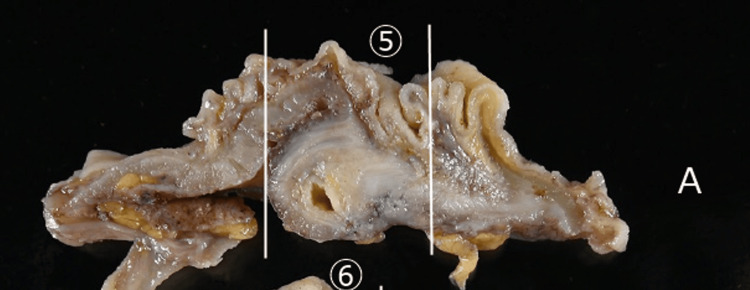
Macroscopic image. Gross image near the Bauhin’s valve showing no grossly visible tumor.

**Figure 4:**
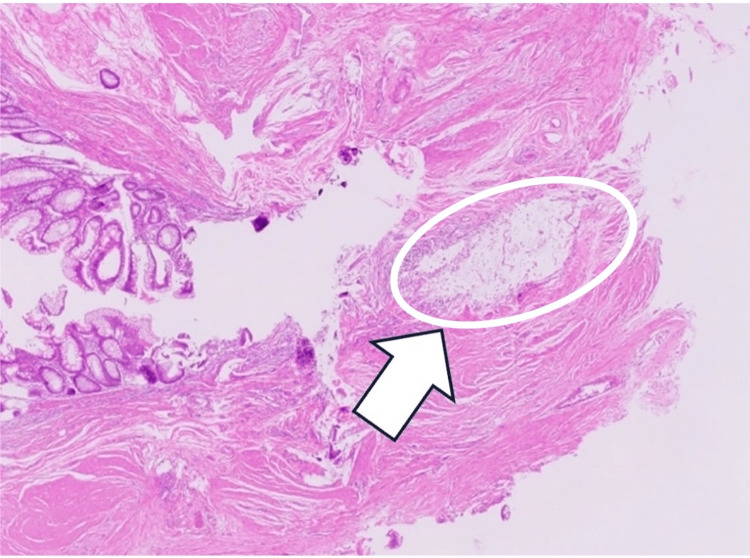
Hematoxylin and eosin (H&E) staining (200x) of the sample collected during the second surgery. Although the cecal surgical margins were negative, discontinuous mucin infiltration of acellular mucinous masses (arrow) was observed in the muscularis propria, deeper than the submucosa, near Bauhin's valve. The mucin infiltration consisted of acellular mucin without identifiable epithelial components and was limited to the muscularis propria.

**Figure 5:**
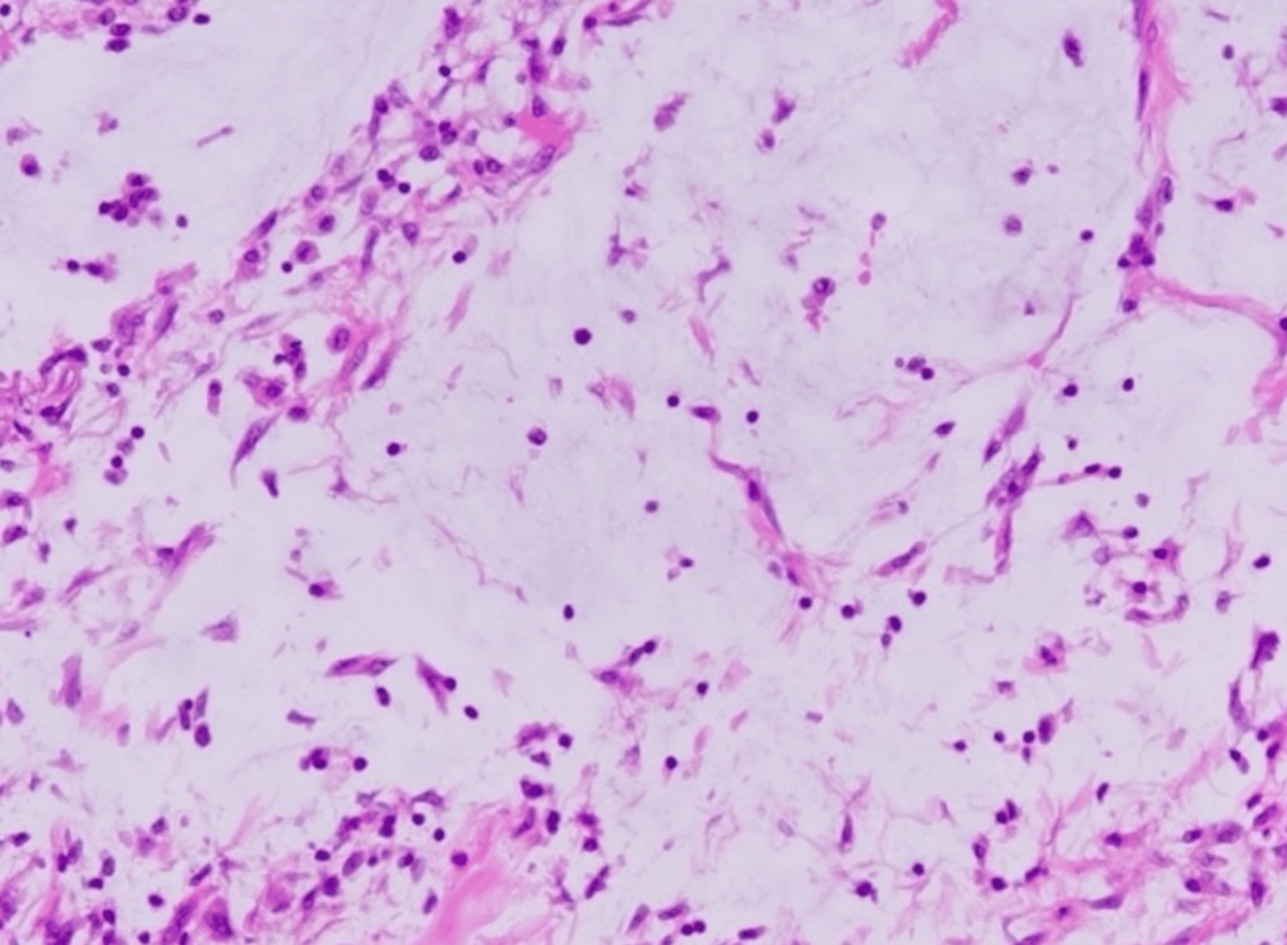
Hematoxylin and eosin (H&E) staining (400x) of the sample collected during the second surgery. No clear epithelial components were observed. Immunostaining results were negative for epithelial membrane antigen (EMA) and cytokeratin (AE1/3, CAM5.2).

Histopathology revealed no macroscopic evidence of infiltration (Figure 3); however, mucin infiltration was observed within the cecal wall near the Bauhin’s valve. The mucin infiltration consisted of acellular mucin without identifiable epithelial components and was confined to the muscularis propria. The surgical margin on the appendectomy side was negative. Immunohistochemical staining for epithelial markers (epithelial membrane antigen (EMA) and cytokeratin AE1/AE3, CAM5.2) was performed to evaluate the presence of epithelial components; however, no epithelial elements were identified, resulting in negative staining (Figures 4, 5).

No obvious lymph node metastases were identified. The mucin infiltration in the cecal wall was discontinuous, with no direct continuity from the appendiceal lesion. Notably, normal tissue was observed between the primary appendiceal lesion and the site of mucin deposition, indicating that this finding was not due to direct extension but rather represented a discontinuous pattern of spread. Pathological examination revealed discontinuous acellular mucin infiltration of the cecal wall, without direct continuity from the appendiceal lesion.

The patient progressed well postoperatively and was discharged on day nine. As pathological findings did not indicate any clear PMP, no additional treatment was performed. Since then, the patient has attended regular follow-up observations for two years postoperatively, with no evidence of recurrence.

## Discussion

The current classification and terminology of LAMN were established by Misdraji et al. in 2003 [4]. Clinically, it is often discovered as a result of abdominal pain or a pelvic mass. In such instances, it is important to distinguish it from appendicitis, adnexal disease, and colon tumors. However, asymptomatic cases are sometimes discovered incidentally based on the finding of painless enlargement of the appendix, as was the case for our patient [5-7].

CT scans are commonly conducted to achieve a preoperative diagnosis. This disease is characterized by an enlarged appendix and low-attenuation intraluminal content due to mucin retention [5,8]. While abscess and tumor formation are sometimes noted [8,9], LAMN can be difficult to diagnose, as some cases only exhibit mild enlargement of the appendix, with a maximum diameter of approximately 10 mm [10].

In the present case, although CT findings suggested LAMN, no clear evidence of mucin infiltration into the cecal wall was identified preoperatively, highlighting the limitations of imaging in detecting discontinuous intramural mucin spread. Retrospective review of the CT images did not reveal any definitive findings suggestive of cecal wall involvement.

Regarding treatment strategies, LAMN rarely causes lymph node metastasis, and lymph node dissection is generally not associated with improved survival rates [11]. However, intraperitoneal dissemination leading to PMP is associated with a significantly worse prognosis, with reported five-year survival rates varying widely depending on disease stage [12]. Dissemination is thought to occur through direct infiltration or perforation of mucus-producing epithelial components.

Although no epithelial components were identified in the present case, both cellular mucinous peritoneal deposits (CMPD) and acellular mucinous peritoneal deposits (AMPD) have been reported [13], suggesting that some cases may develop even without epithelial elements. In rare instances, PMP has been reported to undergo malignant transformation into mucinous carcinoma [10]. Kwak reported that when complete resection is achieved, survival outcomes are excellent (100%), even with limited resection up to the cecum [14]. Therefore, complete resection remains essential in preventing peritoneal dissemination in LAMN.

In the present case, the appendiceal surgical margin was negative, and intraoperative and microscopic findings did not indicate any perforation or peritoneal dissemination. Therefore, this case does not fulfill the diagnostic criteria for PMP.

Despite the absence of intraperitoneal dissemination, discontinuous mucin infiltration was observed in the cecal wall near Bauhin’s valve. This lesion did not fulfill the diagnostic criteria for AMPD. However, it showed some morphological similarities to AMPD, although it should be interpreted cautiously and not considered equivalent.

One possible speculative mechanism for the observed findings is increased intraluminal pressure due to mucin retention. The appendix has a blind-end structure, and its opening can easily become narrowed, potentially leading to pressure elevation. Increased intraluminal pressure may result in mucosal damage or vascular permeation, allowing mucin to infiltrate the wall and contribute to the discontinuous mucin infiltration observed in this case. Similar pressure-related mucosal changes have been suggested in appendiceal disease, although direct evidence in LAMN remains limited.

In cases of LAMN with infiltration of the submucosal layer or deeper, careful consideration of the extent of resection is warranted, as discontinuous mucin infiltration may be present. Further studies are needed to clarify the optimal surgical extent.

To the best of our knowledge, such cases appear to be rare, and reports describing discontinuous mucinous infiltration after secondary resection despite negative surgical margins are limited.

As a limitation, this study is based on a single case, and the follow-up period was limited to two years. Therefore, long-term outcomes remain unclear, and further accumulation of cases with extended follow-up is required.

## Conclusions

In LAMN cases with submucosal or deeper involvement, the extent of resection may require careful consideration, as discontinuous mucinous infiltration could occur. These findings are hypothesis-generating, and further studies are needed to define the optimal surgical strategy.
